# The PSA testing dilemma: GPs' reports of consultations with asymptomatic men: a qualitative study

**DOI:** 10.1186/1471-2296-8-35

**Published:** 2007-06-25

**Authors:** Alison Clements, Eila Watson, Tanvi Rai, Colleen Bukach, Brian Shine, Joan Austoker

**Affiliations:** 1 Cancer Research UK Primary Care Education Research Group, Department of Primary Health Care, University of Oxford. Rosemary Rue Building, Old Road Campus, Headington, Oxford, OX3 7LF, UK; 2 Department of Clinical Biochemistry, John Radcliffe Hospital, Headley Way, Headington, Oxford, OX3 9DU, UK

## Abstract

**Background:**

The National Health Service Prostate Cancer Risk Management Programme (PCRMP) has recommended that screening for prostate cancer is available for asymptomatic men, on the understanding that they have been provided with full and balanced information about the advantages and limitations of the prostate-specific antigen (PSA) test. Guidance has been distributed to all GPs in England and Wales to assist in the provision of information to men. This study aimed to elicit GPs' accounts of their discussions with asymptomatic men who consult with concerns about prostate cancer in order to identify the degree to which the PCRMP guidance was reflected in these consultations.

**Methods:**

Qualitative interview study. Semi-structured telephone interviews with 21 GPs from 18 GP practices in Oxfordshire.

**Results:**

All GPs reported undertaking some discussion with asymptomatic men about the PSA test. They described focussing most of the discussion on the false-positive and false-negative rates of the test, and the risks associated with a prostate biopsy. They reported less discussion of the potential for diagnosing indolent cancers, the dilemmas regarding treatment options for localised prostate cancer and the potential benefits of testing. Considerable variation existed between GPs in their accounts of the degree of detail given, and GP's presentation of information appeared to be affected by their personal views of the PSA test.

**Conclusion:**

The GPs in this study appear to recognise the importance of discussions regarding PSA testing; however, a full and balanced picture of the associated advantages and limitations does not seem to be consistently conveyed. Factors specific to PSA testing which appeared to have an impact on the GPs' discussions were the GP's personal opinions of the PSA test, and the need to counter men's primarily positive views of the benefits of PSA testing. Awareness of the impact of their views on the consultations may help GPs give men a more balanced presentation of the benefits and limitations of the PSA test.

## Background

Screening for prostate cancer is controversial. The high incidence and mortality rates [1], and the fact that localised disease can potentially be treated, has led to widespread calls for prostate cancer screening. However, there is ongoing debate as to whether screening would result in more harm than good [2,3]. There is no strong evidence that screening would result in reduced mortality from the disease [4,5]. The PSA test has relatively high false positive and false negative rates, prostate biopsies can miss cancers, and the highly variable nature of prostate cancer means there is potential for diagnosis of indolent cancers that may never present as a problem [6,7].

In the UK, the National Screening Committee has recommended that a prostate cancer screening programme should not be introduced [8]. However, in response to growing public concern about the disease, in 2001 the Department of Health introduced the Prostate Cancer Risk Management Programme (PCRMP). Central to this programme is the recommendation that *any man who wishes to have a PSA test should have access to the test, provided he has been given full information regarding the possible benefits and limitations associated with receiving a test *[9]. A national information pack was developed to provide guidance for primary care in providing men, who ask their GPs about testing for prostate cancer, with clear and balanced information on the benefits and limitations of testing [10]. The key information points are summarised in Table 1.

**Table 1:** Key points identified by the PCRMP for men to be aware of prior to undertaking a PSA test

• the PSA test facilitates the early detection of prostate cancer at a stage when potentially curative treatments can be offered
• there is currently no strong evidence that PSA testing reduces mortality from prostate cancer
• not all men with raised PSA will have prostate cancer/the PSA test will not detect all prostate cancers
• prostate cancer is diagnosed through a prostate biopsy which can be uncomfortable or painful
• prostate biopsies will not detect all prostate cancers
• prostate cancers range from aggressive to slow growing forms – slow growing tumours may not result in symptoms or shorten life expectancy
• there is no evidence about the optimum treatment for localised prostate cancer
• some treatments for prostate cancer can have significant side effects

It is not known to what extent primary care consultations about PSA testing reflect the guidance provided by the PCRMP. Our study sought to understand GPs' interactions with men who consult them without having any of the symptoms associated with the disease (asymptomatic men) and to identify the degree to which the PCRMP guidance was reflected in the consultations. This paper describes the discussions that GP's report having with these men, as well as factors that appear to influence the nature and content of the discussions. The discussion considers the extent to which it may be possible for GPs to follow the PCRMP guidance within the consultation.

## Methods

### Recruitment

A purposive sample of GPs was identified through first PSA test requests made for patients, of any age, to the Department of Clinical Biochemistry, John Radcliffe Hospital, Oxford. As part of a separate study, questionnaires had been sent to the requesting GPs. Of the 173 GPs who returned a questionnaire, 94 indicated that they would be willing to also take part in a telephone interview. Consecutive GPs were invited to take part in this study. 21 GPs, from 18 surgeries, were interviewed within the time frame of the study. GPs were paid £50 as reimbursement for the time spent. See Table 2 for the characteristics of recruited GPs.

**Table 2:** Characteristics of recruited GPs

	**n**	**%**
**Gender**		
Male	15	71.4
Female	6	28.6
		
**Time practising (years)**		
<5	3	14.3
5–15	6	28.6
16–25	7	33.3
>25	5	23.8
		
**Age (years)**		
30–39	6	28.6
40–49	6	28.6

### Data collection

Semi-structured telephone interviews were conducted by TR and EW. An interview guide was used to elicit i) the content of discussions GPs have with asymptomatic men who consult with concerns about prostate cancer/PSA testing and ii) the attitudes of GPs toward the PSA test. Other areas were covered but are not the topic of this paper. With permission the interviews were tape-recorded and fully transcribed.

### Analysis

Data analysis was undertaken by AC, TR & EW using the framework approach [11]. Through reading and re-reading the data, an analytic framework was developed from the identification of the key issues within the data (using a priori issues and questions from the aims of the study, in addition to issues raised by the participants). This coding framework was used to analyse all data, with themes added as new issues were identified in subsequent stages of the analysis. A transparent coding scheme, together with regular discussions between the researchers during the coding and interpretation stages helped to ensure the credibility and trustworthiness of the findings [12]. Data across the whole set as well as within each individual interview were examined. The software package, Atlas.ti, was used to assist in the management of the data.

## Results

All GPs in the study reported having some degree of discussion about PSA testing with asymptomatic men who consulted with concerns about prostate cancer. However, we found considerable variation within the reported discussions, with a tendency for greater emphasis to be placed on certain key points and disparity in the degree of detail given. We also identified differences in the impartiality with which GPs appeared to present the information.

### Content of GPs' discussions with men

### False-positive and false-negative results

The GPs in our study described feeling that it was important for men to understand the imprecise nature of the PSA test, and, without exception, they reported having discussed the possibility of the PSA test yielding false-positive and false-negative results.

"I normally tell them that men with prostate cancer usually have high levels of PSA and men without prostate cancer usually have low levels but there are some men who have higher than normal levels who don't have prostate cancer and some men who've got quite low levels who turn out that they do have prostate cancer..." ID19

### The biopsy stage

Many GPs said they would explain that the PSA test alone is not sufficient to diagnose prostate cancer and a prostate biopsy is the probable next stage following a raised PSA result.

"...it's usually part of my talk that ... if it is positive then the only way we go further and know for sure would be a biopsy..." ID10

However, the uncertainty of what to do if the PSA result is only slightly raised, is also often discussed.

"...*[I talked] *about the false-positives and negatives, and the problem of marginally raised results, what to do with it. The difficulties in deciding what to do with a slightly raised PSA ....." ID3

GPs frequently reported discussing the potential pain and discomfort of a biopsy. Less often they described mentioning that some biopsies are unwarranted due to false-positive PSA results, and that false reassurance can result from false-negative biopsy results.

"...*[I say that] *it's not a very good test and it's, there's a fair chance that it'll show up positive and you won't have any prostate cancer ... so that can land you in all sorts of unpleasant biopsies and things for nothing." ID17

While many GPs reported that they would discuss issues related to a prostate biopsy, there was considerable variation in the degree of detail that they said they give. Some would provide minimal information.

*"...do you actually mention the possibility of having to have a biopsy if the PSA is raised ...? *I'm not sure I get as far, I would say "If it's raised and it's raised enough that we need to refer you for more investigations," and I think that would be as far I would get..."ID2

Others report giving much more expansive descriptions.

"... then I go on a bit to talk about biopsies... that when they do an ultrasound test they can sometimes see an area of abnormality in which case they can biopsy that area..... but if they can't see any areas of abnormality then they take six or eight blind biopsies and the results of those biopsies can be again normal or abnormal. And when they're normal then that's usually reassuring but of course if you're taking blind biopsies you may have missed the area of abnormality......and so it can never be absolutely foolproof ..." ID18

### Potential for diagnosis of indolent cancers

While less frequently discussed than the previous key points, a number of GPs said they would discuss the variable nature of prostate cancer, with an emphasis on the possibility of identifying indolent cancers which may never cause a problem.

"... my usual line is, when old boys die an awful lot of them have been found to have prostate cancer that they had no idea was there and has never caused a jot of problems...and I try and explain that to them 'You may turn out to have the prostate cancer that is just going to sit there for thirty years and do nothing...' " ID15

GPs who said that they either would not include a discussion of the range and nature of prostate cancers, or would do so in a very limited way, gave several reasons for this. Some felt that this information is often not relevant to men at this stage of the process.

"I feel I'm in second line for that (*discussion of range of prostate cancers*) because if they go ahead and have the biopsy, say they have the PSA test and it's positive then you have to, you are obliged to refer ... the urologists are obliged then to investigate further.....so it's very much their stance isn't it?" ID8

Some GPs believe this information is less relevant for younger men, as having prostate cancer at an early age is more likely to have serious implications.

"I think it's useful in the elderly...but I don't think it's useful in someone aged 50...because they, if they did get diagnosed with prostate cancer at 50 then they probably would die of it..." ID16

### Treatments for prostate cancer

Very few GPs in the study reported discussing treatment options for early prostate cancer. Those who did said they would tend to emphasise the potential side effects and lack of consensus about which treatments, if any at all, may be effective.

"I think people need to realise ...even if it is diagnosed, as far as I know there is still uncertainty as to what the best treatment is even so if you know you've got it, it's hard to know what to do about it" ID19

Again, relevance of the information to men at this stage of the process was cited as a reason for omitting treatment option discussions.

"you only have a limited consultation ...you don't want to frighten people if they haven't got it, like you don't say to every woman before they go for a mammogram all the pros and cons of chemotherapy and radiotherapy for breast cancer do you?" ID7

GPs reported very little corresponding discussion about how treatment could be potentially beneficial.

"... I don't go into all the detail about what the various treatment options are... just to say that there are effective treatments particularly if it's caught early which is the rationale really for screening for it". ID14

Several factors which had an impact on what was discussed within the consultations are specific to the PSA test and have been considered in the relevant sections above; others factors relate to primary care consultations in general.

### Written information

The amount of detail discussed was affected by the use of written information. Some GPs found patient information leaflets a useful supplement to, or in some cases, a replacement for detailed discussion.

-"*...so you tend to give the leaflet out? *Oh always. *Do you think that is the best way to provide patient information, for GPs? *Um well I think the evidence is that people don't remember what you say to them ... and it also means that you know wives and families generally have a chance to share it, so I know I'm also, everything that can be given in leaflet form should be really" ID13

### Time factors

Explaining and ensuring an understanding of the key points was felt by many GPs to be a time consuming process. Some felt that a ten minute consultation, focussing only on the PSA test, may give sufficient time to convey the main points, though this would not be so if more than one concern is brought to a consultation and the issue of PSA testing raised at a late stage. Other GPs felt that even a full 10 minute consultation would not be sufficient.

*"I don't know if our language has developed enough with patients nor their, nor their thoughts. I think, I think they're difficult concepts.....So I think, I think getting a patient to, to a position of truly informed decision-making is, is difficult and it is very time consuming and maybe that's the problem, maybe I just feel we haven't got the time to spend with every patient doing that" *ID5

### Degree of balance in discussion

### GPs' opinions about the PSA test

We found differences in the way GPs described how they present information to men, which in part seems to reflect the wide range of opinions held about the usefulness of the PSA test as a screening tool. Some GPs who expressed strong views against the value of the PSA test, quite clearly portray this view to men in their discussions, either directly:

" ...I think I would really give very directive counselling and try and talk them out of it and say "It's a waste of time and really I advise against it." ID17

or indirectly, by highlighting the drawbacks:

"... I do tend to talk about the impossible situation you get yourself into if you have a PSA that's raised. You're going to then not know whether to have prostate biopsies and so on... and all the risks of that... so that's how I tend to try to put them off..... by saying 'The chances are it will come back in some unhelpful grey area and you won't know what to do...' " ID13

GPs who have either neutral, or more positive views about the usefulness of PSA screening tend to present men with information on the benefits and limitations of testing, with the intent of allowing the men to reach their own decisions. One such GP, who provided very full information on testing, did note that most men he counselled went on to be tested.

"I'm probably a bit pro-testing ... I don't positively dissuade them as it were, I give them all the information and they usually go on to have a test, I would say ...". ID18

### Men's prior opinions of PSA testing

Men's predominantly positive attitudes towards PSA testing, often due to press coverage or personal recommendation, also often had an effect on the way GP's presented the information. GPs described emphasising the drawbacks of testing, thus presenting somewhat biased information to counter the faith men appeared to place in the test.

"...and people in general are quite keen to have, men in general are quite keen to have a prostate blood test.....unfortunately... *[laughs]*... I certainly sound negative about it to counteract the over-positive things that they've read ..." ID4

### Impact of balance on GP's behaviour

While personal views about PSA testing seem to be an important factor in determining how impartial GPs are in presenting information, and many GPs did say they would attempt to dissuade men from being tested, only one GP in our study said he would deny a man's request, and that related specifically to men under the age of 50. Concerns about being held responsible for missing a cancer affected the tendency for some GPs to dissuade a man from undergoing a PSA test. The following GP said he would highlight the negative elements of testing to men, but would carry out a test if they persisted in their request.

"...people have read about it (*PSA test*) and are convinced it's the bees knees ...and if you don't do it and it turns out that subsequently you could have detected prostate cancer I think you know you, you're going to be on a sticky wicket..." ID15

## Discussion

### Summary of findings

While GPs' descriptions of their consultations suggest that they recognise the importance of discussions with asymptomatic men about the PSA test, a full and balanced picture of the associated benefits and limitations does not appear to be consistently conveyed. GPs reported talking about some of the limitations of the PSA test: the false-negative and false-positive results and the potentially unpleasant nature of a prostate biopsy, while much less so about the potential for identification of indolent cancers and the lack of evidence regarding treatment effectiveness. Relatively little attention seems to be given to the potential benefit of PSA testing: the identification of early prostate cancer which could be successfully treated. However, there was considerable diversity in the degree of detail GPs reported conveying to men about these points. The GPs' personal opinions of the PSA test, and their perceived need to counter men's primarily positive views of the benefits of PSA testing appeared to have an impact on the GPs' discussions.

We have identified both practical and ideological barriers faced by GPs in providing a full and balanced picture of the benefits and limitations of the PSA test. Constraints such as consultation time, could be addressed by the consistent provision of written information, together with a second consultation prior to testing. More complex to address may be the impact of GPs' views on the usefulness of the PSA test and their feelings of the relevance of specific information at particular times.

Providing information to patients about clinical issues for which there is no established evidence base can be problematic, and it is understandable that GPs' discussions could reflect their clinical experience and personal opinion. The views held by many GPs about the value, or lack of value of the PSA test appear to be a driving force behind the information they give and how it is presented. Support for this finding comes from work in the US. Cooper has shown that views held, both for and against PSA testing, influence guideline adherence. Physicians holding positive views tended to screen routinely with little accompanying discussion, while those influenced by the lack of evidence of benefit, and their personal and professional experiences of the limitations of the PSA test were more likely to discuss the implications of testing [13]. Work by Chan also suggested a belief that the benefits of testing outweigh the risks may create a bias in the discussion of PSA testing with patients [14]. It may be that the views of GPs regarding the value of the PSA test will be affected by evidence from two large-scale randomised screening trials being conducted in Europe and the USA, aiming to assess whether PSA screening reduces prostate cancer mortality [15]. Trial results are expected in 2008 and should enable definitive recommendations about the value of PSA screening to be made. This will change the position GPs are currently in, of being expected to provide information without good evidence of the impact of PSA testing on mortality. However, while a national screening programme may follow if the trials do provide evidence of mortality reduction, the development and implementation of such a programme is likely to take several years. In the meantime, information about the benefits and limitations of testing will still need to be portrayed to men, and it is important that how GPs interpret and incorporate new findings into their consultations continue to be understood.

Our findings indicate that a GP's perception of the relevance of having certain information while deciding whether to undergo a test may also have an impact on the balance of the discussions. It is interesting that GPs reported being less likely to discuss the potential for diagnosing indolent cancers and the lack of evidence for the effectiveness of prostate cancer treatments, as these two points are often cited as reasons against introducing population screening [16,17]. On the other hand, it has been proposed that it may be misleading to focus on the potential for over-diagnosis when discussing screening with younger men [18]. Other factors, such as the complexity of conveying concepts of risk, responding to personal knowledge of their individual patients and an awareness that not all men want information may also be important determinants in shaping the discussion.

The lack of balance that some GPs report in their discussions can also be understood in terms of what they see as men's over-positive views of the PSA test. This is not an issue peculiar to prostate cancer screening; as with many screening tests, the primary benefit to the patient is detection of curable disease, which to an individual, may outweigh the greater number of potential drawbacks. The apparent concentration on the test's limitations may be an attempt by GPs to counter and bring a sense of balance to the men's views. However, the impact of this may be that the potential benefits of PSA testing are not discussed, but left implicit, possibly creating an imbalance in the opposite direction.

It may help GPs to be conscious of the impact that their views of the PSA test and their perceptions of men's need for information may have on their consultations. Incorporation of our findings in a revision of the PCRMP information pack will hopefully enable GPs to present information on PSA testing to men in a consistently balanced way.

### Strengths and limitations

This study is the first to address the discussions about PSA testing that take place during GP consultations with asymptomatic men concerned about prostate cancer, and as such provides a valuable insight into the extent to which the implications of undergoing a PSA test are discussed. The value of qualitative research lies in the depth of understanding gained from detailed descriptions of specific experiences. For this reason the number of participants in a qualitative study is necessarily small. However, we do acknowledge that the relatively small number of GPs interviewed from those available may mean there were a range of experiences that we have not captured. Interviews reliant on recollections of an event can suffer from recall bias. While audio-taped consultations would have provided an accurate record of the actual consultation, the infrequency of relevant consultations and the complexity of determining in advance that the topic would be discussed and obtaining consent made this approach unfeasible. A further limitation is that GPs for this study were recruited from one regional area. However, given the wide range of behaviours and views described, and that very few GPs' accounts reflected a 'model' consultation according to the guidance offered, there is nothing to strongly suggest that our data collection methods or recruitment strategy have limited our findings.

### Implications for future research

Extending this initial work through the use of observation methods may add to our understanding of consultations, though the methodological difficulties inherent in such a study should not be underestimated. Furthermore, while the GPs in our study indicated they would not deny a man over 50 a PSA test, we do not know how men are affected by the discussions they have with their GP. It is important to understand men's perception of the information they are given, whether they want it and what use of it they make. We have completed an interview study looking at consultations prior to PSA testing from men's perspective, which will be reported shortly.

## Conclusion

The GPs in this study appear to recognise the importance of discussions regarding PSA testing; however, a full and balanced picture of the associated advantages and limitations does not seem to be consistently conveyed. Factors specific to PSA testing which appeared to have an impact on the GPs' discussions were the GP's personal opinions of the PSA test, and the need to counter men's primarily positive views of the benefits of PSA testing. Awareness of the impact of their views on the consultations may help GPs give men a more balanced presentation of the benefits and limitations of the PSA test.

## Competing interests

The author(s) declare that they have no competing interests.

## Authors' contributions

AC supervised the data collection, analysis and interpretation of data and drafted the manuscript

EW conceived of the study, participated in the data collection, analysis and interpretation of data and helped to draft the manuscript

TR participated in the data collection, analysis and interpretation of data and helped to draft the manuscript

CB participated in the design of the study, data collection, analysis and interpretation of data and the revision of the manuscript

BS participated in the design of the study, the data collection and the revision of the manuscript

JA conceived of the study and participated in the revision of the manuscript

All authors read and approved the final manuscript

## Pre-publication history

The pre-publication history for this paper can be accessed here:


